# Dietary supplements for Parkinson's disease: State of the science

**DOI:** 10.1177/1877718X261446386

**Published:** 2026-05-13

**Authors:** Aakash Prasad, Marshal Spenser Shuler, Richelle Flanagan, Viswas Dayal, Fiona E Lithander

**Affiliations:** 1 Liggins Institute, University of Auckland, Auckland, New Zealand; 2 My Moves Matter, Dublin, Ireland; 3 Neurology Department, Auckland City Hospital, Auckland, New Zealand

**Keywords:** Parkinson's disease, dietary supplements, clinical trials, disease modification

## Abstract

Parkinson's disease (PD) is the fastest growing neurological condition worldwide with its prevalence set to double by 2050. With no cure in sight, management has turned to lifestyle modification, in particular to diet and exercise. The disease-modifying potential of dietary approaches has been of recent interest, particularly given emerging links between diet and reductions in systemic inflammation, oxidative stress, and alterations in the gut microbiome composition, all of which may modulate neurodegeneration. This review summarises the current ‘state of the science’ of dietary supplements in modifying disease progression through a lens of the pathophysiological hallmarks of PD. Biomarkers and clinical outcomes that serve as proxy measurements for disease modification are examined, whilst looking ahead at which dietary supplements show the most promise and should be the focus of future research.

## Plain language summary

The number of people with Parkinson's disease will grow over the next few decades. Despite years of trialing different pharmaceuticals, a cure remains to be found. For this reason, lifestyle has gained more attention in recent years. Current research suggests that regular physical activity may have the potential to slow the progression of the disease. A growing number of clinical trials have examined how different dietary approaches can alter Parkinson's. These include trials of dietary patterns, foods, nutrients and dietary supplements. In this article, an overview of the current evidence for dietary supplements and their effects on Parkinson's disease progression is provided. The dietary supplements that currently hold the most promise for forthcoming research are highlighted, and future directions within the field are discussed.

## Introduction

Since the 1960s, the gold standard treatment for Parkinson's disease (PD) has remained levodopa therapy. Although highly effective for managing motor symptoms, the emergence of motor fluctuations and levodopa related complications due to the degenerative nature of PD limit its effectiveness as the disease progresses.^1^ In order to slow or stop disease progression, interventions must target the pathogenic steps that ultimately drive dopaminergic neuronal loss within the substantia nigra pars compacta of the basal ganglia, as well as the more widespread loss of neurons in the brainstem and cortical regions. PD is characterised by several core pathophysiological hallmarks including *α*-synuclein misfolding and aggregation, impaired protein clearance, mitochondrial dysfunction, and neuroinflammation and oxidative stress.^2^ For example, circulating inflammatory biomarkers, including tumour necrosis factor-*α* (TNF-*α*), interferon-y (IFN-y) and various interleukins are elevated in PD relative to control patients.^3^ More recently, additional pathophysiological hallmarks, such as gut microbiome dysbiosis,^4^ endoplasmic reticulum impairment^5^ and neuronal excitotoxicity^6^ have been proposed. All of these hallmarks are closely interlinked and correlate positively with nigral neurodegeneration.^7^

**Figure 1. fig1-1877718X261446386:**
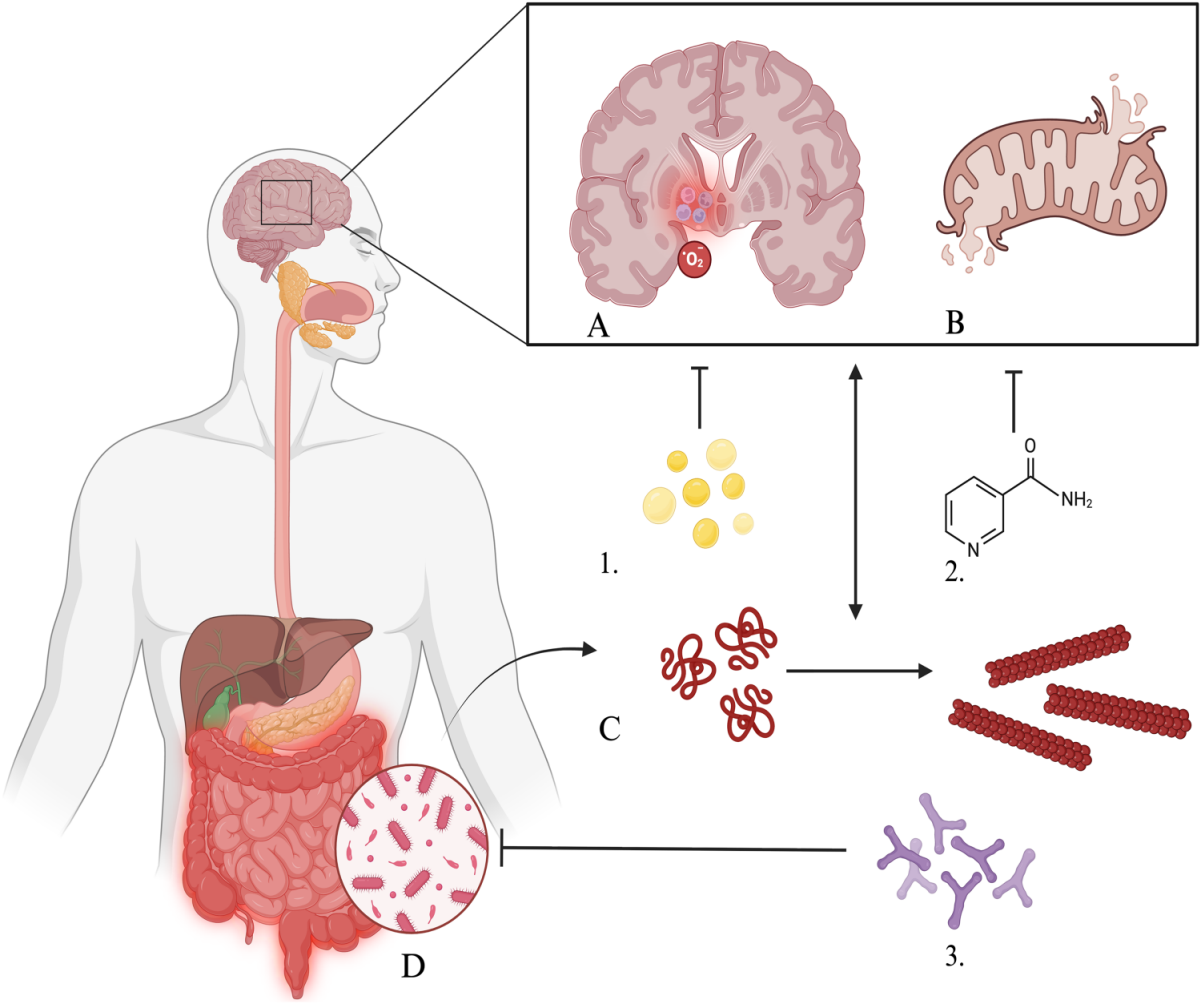
Primary mechanisms by which omega-3 fatty acids (*n*-3), nicotinamide riboside, and biotics influence key pathophysiological hallmarks of Parkinson’s disease. Inflammation, oxidative stress, and mitochondrial dysfunction are core, interconnected hallmarks that interact bidirectionally with α-synuclein aggregation. *n*-3 and NR may attenuate inflammation, oxidative stress, and mitochondrial dysfunction, whereas biotics modulate the gut microbiome, influencing α-synuclein aggregation and downstream pathways. Pathophysiological hallmarks: A, Neuroinflammation and oxidative stress; B, Mitochondrial dysfunction; C, α-synuclein aggregation; D, Gut dysbiosis. Interventions: 1, *n*-3; 2, NR; 3, Biotics.

Multiple phase II and III trials have examined pharmacological agents aimed at modulating these pathophysiological hallmarks; however, all have failed to demonstrate significant disease-modifying effects.^8^ Therefore, due to challenges in determining disease-modifying pharmacological therapies, research focus has increasingly shifted towards lifestyle, including diet and exercise.^9–11^ Whilst the positive effect of exercise is now well-established, with strong evidence supporting its neuroprotective and disease-modifying potential,^12,13^ evidence from trials of diet, including dietary supplements, remains limited. Mechanistically, diet offers plausible benefits for neuroprotection and disease modification due to potential antioxidant and anti-inflammatory properties,^14^ as well as potential positive effects on the gut microbiome.^15^ Dietary supplements are of particular interest in PD, as their primary function is to add to or supplement^16^ the diet, making them convenient to administer and well suited for clinical trials, where participants can be effectively blinded. Furthermore, in the studies that have investigated supplement use in PD, up to two thirds of participants reported using dietary supplements.^17,18^ In addition, similar to dietary interventions, several studies suggest that certain dietary supplements possess relevant mechanisms of action thought to be able to decelerate or even stop the pathophysiological processes driving PD, including neurodegeneration, *α*-synuclein pathology, oxidative stress, inflammation, mitochondrial dysfunction and gut microbiome dysbiosis. Examples of dietary supplements include vitamins, minerals, botanicals, herbs, amino acids, biotics^16^ and other such products. While both *in vitro* and *in vivo* studies have established the therapeutic potential of dietary supplements, human clinical trials are required to determine causality between a particular dietary supplement and PD modification.[Fig fig1-1877718X261446386]

Alongside biomarker-based assessment of the pathophysiological hallmarks, disease progression in trials is often clinically measured using the Movement Disorder Society-sponsored Unified Parkinson's Disease Rating Scale (MDS-UPDRS) and the Hoehn and Yahr (H&Y) staging scale. Both the total MDS-UPDRS score and the H&Y scale are used to measure disease progression and severity.^19,20^

The aim of this review is to summarise the current state of the science and future research perspectives regarding dietary supplements with a focus on their potential to modulate PD pathophysiological hallmarks and clinical disease progression. As a result, only human clinical intervention trials are discussed. Importantly, this review distinguishes between dietary supplements used for the purpose of correcting biochemical deficiencies and those explored for disease-modifying potential. The former, such as supplementing vitamin D for bone health or vitamin B12 for neuropathy, although remaining clinically important when circulating levels are low, are outside the scope of this review. Instead, the focus is on dietary supplementation administered beyond physiological replacement, with the aim of altering PD progression. This distinction is particularly important given the high prevalence of supplement use among PD patients.^17,18^ Understanding the current evidence base is essential to help clinicians and patients make informed decisions regarding supplement use.

## Dietary supplements

Multiple studies have investigated the effect of dietary supplements on the clinical progression of PD. This section describes the findings from clinical trials for all dietary supplements evaluated to date. The databases MEDLINE, CINAHL, and EMBASE were searched for relevant trials and references from primary papers were also examined. The inclusion criteria for a trial to be considered for this review were: human clinical trial; examined the effects of a dietary supplement(s); assessed disease modification through the use of UPDRS-total and/or H&Y clinical scales and/or through biomarker-based assessment of the pathophysiological hallmarks. The search yielded 38 trials which have investigated the effect of dietary supplements including omega-3 fatty acids, vitamins B, D and E, compounds such as creatine, coenzyme Q10, inosine and curcumin, and biotics on disease modification (Table 1).

**Table 1.** Overview of clinical intervention trials that assessed the disease modifying potential of dietary supplements.

Intervention	Trials (total)	Outcomes	Measurement tools/biomarkers	Findings^a^
Omega-3 fatty acids	5	Clinical disease progression	UPDRS-total	Conflicting findings (2 trials: 1 positive, 1 negative)
			Hoehn and Yahr	No significant between-group improvement (3 trials)
		Inflammation	hs-CRP (blood)	Significant between-group improvement (1 trial)
			TNF-*α*, IL-1, IL-8 (gene expression)	Significant between-group improvement in TNF-*α* (1 trial)
		Oxidative stress	TAC, MDA, GSH, NO (blood)	Significant between-group improvement in TAC and GSH (1 trial)
B-vitamins	6	Clinical disease progression	UPDRS-total	Conflicting findings (3 trials: 1 positive, 2 negative)
			Hoehn and Yahr	No significant between-group improvement (2 trials)
		Inflammation	Inflammatory cytokines (blood)	No significant between-group improvement (2 trial)
			Immune cells (gene expression & blood)	No significant between-group improvement (3 trials)
		Mitochondrial function	GDF15 & FGF21 (blood & CSF)	No significant between-group improvement (1 trial)
		Neuroaxonal damage	Neuroﬁlament light chain (blood & CSF)	No significant between-group improvement (1 trial)
		Oxidative stress	NAD & glutathione metabolites (cerebral, peripheral tissues, CSF, blood & urine)	Conflicting findings (4 trials)
Vitamin D	3	Clinical disease progression	UPDRS-total	No significant between-group improvement (2 trials)
			Hoehn and Yahr	Significant between-group improvement (1 trial)
		Inflammation	hs-CRP (blood)	No significant between-group improvement (1 trial)
Vitamin E	1	Clinical disease progression	UPDRS-total	No significant between-group improvement (1 trial)
			Hoehn and Yahr	
Creatine	3	Clinical disease progression	UPDRS-total	Conflicting findings (3 trials: 1 positive, 2 negative)
		Neuroaxonal damage	Dopamine transporter density (SPECT imaging)	No significant between-group improvement (1 trial)
Coenzyme Q10	7	Clinical disease progression	UPDRS-total	Conflicting findings (6 trials: 2 positive, 3 negative, 1 miscellaneous)
			Hoehn and Yahr	Conflicting findings (5 trials: 1 positive, 4 negative)
		Mitochondrial function	Mitochondrial complex I/III activity	Significant between-group improvement (1 trial)
		Oxidative stress	Oxidative damage markers & antioxidants (blood)	No significant between-group improvement (1 trial)
Inosine	2	Clinical disease progression	UPDRS-total	Conflicting findings (2 trials: 1 positive, 1 negative)
			Hoehn and Yahr	No significant between-group improvement (1 trial)
		Neuroaxonal damage	Dopamine transporter density (SPECT imaging)	No significant between-group improvement (1 trial)
Curcumin	1	Clinical disease progression	UPDRS-total	No significant between-group improvement (1 trial)
			Hoehn and Yahr	
Probiotics	7	Clinical disease progression	UPDRS-total	Conflicting findings (2 trials: 1 positive, 1 negative)
		Gut microbiome	Taxonomic gene profiling (feces)	Conflicting findings (4 trials: 3 positive, 1 negative)
		Inflammation	TNF-*α*, IL-1, IL-8, TGF-β (gene expression)	Significant between-group improvement in TNF-*α*, IL-1, IL-8, TGF-β (1 trial)
			Inflammatory cytokines, hs-CRP (blood)	Significant between-group improvement in TNF-*α*, IL-1β, INF-*γ*, IL-6, IL-10, and hs-CRP (3 trials)
		Oxidative stress	TAC, GSH, MDA, NO (blood)	Significant between-group improvement in MDA and TAC (2 trials)
Prebiotics	1	Clinical disease progression	UPDRS-total	No significant between-group improvement (1 trial)
		Gut microbiome	Taxonomic gene profiling (feces)	No significant between-group improvement (1 trial)
		Inflammation	Zonulin, calprotectin, CRP, inflammatory cytokines (blood)	No significant between-group improvement (1 trial)
Synbiotics	4	*α*-synuclein pathology	*α*-synuclein (blood)	No significant between-group improvement (1 trial)
		Clinical disease progression	Hoehn and Yahr	No significant between-group improvement (1 trial)
		Gut microbiome	Taxonomic gene profiling (feces)	Significant between-group improvement (1 trial)
		Inflammation	TNF-*α*, IFN-*γ*, IL-6, TGF-β (blood)	Conflicting findings (2 trials)
		Oxidative stress	TAC, TOS, GSH, MDA, OSI (blood)	Significant between-group improvement in TAC, OSI and MDA (2 trials)

^a^ Only between-group analyses reported.

Abbreviations: CSF, Cerebrospinal fluid; GSH, glutathione; hs-CRP, high-sensitivity C-reactive protein; IFN, interferon; IL, interleukin; MDA, malondialdehyde; NO, nitric oxide; OSI, oxidative stress index score; SPECT, Single Photon Emission Computed Tomography; TAC, Total Antioxidant Capacity; TGF, transforming growth factor; TNF, tumor necrosis factor; TOS, Total Oxidant Status; UPDRS, Unified Parkinson's Disease Rating Scale.

### Omega-3 fatty acids

The importance of adequate omega-3 (*n*-3) fatty acid intake is well-established for cardiovascular benefits^21^; however, emerging evidence suggests they may also exert neuroprotective effects in PD by modulating inflammation and oxidative stress, increasing levels of brain-derived neurotrophic factor and inhibiting apoptosis.^22,23^ Five randomised controlled trials (RCTs)^24–28^ investigated the effects of *n*-3 fatty acids on either clinical measures of disease progression or pathophysiological biomarkers of PD, including inflammatory and oxidative stress biomarkers. Four of these trials, however, did not use *n*-3 fatty acids as a monotherapy. One trial^25^ evaluated *n*-3 fatty acids alone, while 3 of the remaining 4 RCTs combined *n*-3 fatty acid supplementation with vitamin E. Pantzaris et al.^26^ instead administered “Neuroaspis”, a supplement composed of the *n*-3 fatty acids eicosapentaenoic acid and docosahexaenoic acid, *γ*-linolenic acid, linoleic acid, vitamin A, vitamin E and *γ*-tocopherol. As a result, it is difficult to attribute observed effects to *n*-3 fatty acids alone, as synergistic or complementary actions between nutrients can occur.

With respect to clinical outcomes, findings across trials are inconsistent, likely reflecting differences in study methodology, design, sample size and supplementation protocols. Three RCTs^24–26^ investigated H&Y stage, yet none demonstrated significant benefits of *n*-3 fatty acid supplementation relative to placebo, whether administered alone, combined with vitamin E, or as part of the Neuroaspis formulation. While Tamataji et al.^28^ reported no significant changes in UPDRS total score, an RCT with the same design, supplementation regime and duration by Taghizadeh et al.^27^ found significant improvements following supplementation. Given the similarities between the 2 RCTs, it is possible that the larger sample size of Taghizadeh et al.^27^ contributed to the statistical significance of its UPDRS findings. Furthermore, total UPDRS score was designated as the primary outcome in the Taghizadeh et al.^27^ trial, whereas Tamataji et al.^28^ focused primarily on inflammatory biomarkers. Thus, the latter trial was not powered to detect changes in UPDRS to the same extent.

The same 2 trials^27,28^ were also the only trials to examine pathophysiological biomarkers, with both demonstrating reductions in inflammatory and oxidative stress parameters following *n*-3 fatty acids combined with vitamin E when compared to placebo. Such findings suggest possible disease-modifying potential. It is conceivable that a longer trial duration may better clarify the impact of *n*-3 fatty acids on clinical endpoints. Thus, *n*-3 fatty acid supplementation remains a promising candidate for further investigation in larger, more rigorously designed trials.

### B-vitamins

B-vitamins, comprising 8 individual vitamins, play essential roles in cellular metabolism, acting as cofactors for numerous enzymatic reactions and supporting mitochondrial function.^29^ Given the limited available evidence from 6 trials, there is conflicting data on the promise of B-vitamins as disease-modifying supplements in PD. Clinical improvement, measured using the MDS-UPDRS total score, served as a secondary outcome in both the NADPARK^30^ and NR-SAFE^31^ trials, each of which administered nicotinamide riboside (NR), a form of vitamin B_3_. In the phase I NADPARK trial which involved 30 PD participants over 30 days, NR supplementation failed to show any improvements in slowing disease progression. Alongside these negative clinical findings, several pathophysiological biomarkers, including pro-inflammatory cytokines, decreased to the same extent in both intervention and placebo groups.^30^ Subsequently, the NR-SAFE trial was conducted by the same investigators and enrolled 20 PD participants for 28 days and reported a significant reduction in total MDS-UPDRS score, largely driven by motor improvement measured using Part III^31^ The discrepancy between these two trials may be partly attributable to their differing dosages, whereby the NADPARK trial administered 1000 mg/d compared to 3000 mg/d in NR-SAFE.^30,31^ Two additional RCTs investigating NR are ongoing, with results expected in the coming years. Another recently published trial assessed “combined metabolic activators” (CMA), a mixture including serine, NR, N-acetylcysteine and carnitine tartrate on PD progression; however, this intervention did not reduce total UPDRS score over 84 days,^32^ suggesting that combining NR with other metabolic cofactors does not necessarily yield added clinical benefit.

Beyond NR, other B-vitamins have been minimally explored in the context of disease modification. Lee et al.^33^ assessed a combination of vitamins B_9_ and B_12_ over 12 months using H&Y staging as an explanatory outcome, and reported no significant differences in H&Y between the two groups. DiFrancisco-Donoghue and colleagues,^34^ on the other hand, evaluated the 6-week effect of vitamins B6, B9, and B12 as a standalone treatment or in combination with exercise on oxidative stress markers. The study found that only supplementation with exercise, or exercise alone, positively affected circulating levels of glutathione and its metabolites. Chong et al.^35^ observed no between-group differences following the 12-week supplementation of niacin (vitamin B_3_) on inflammatory markers. However, the 12-month open-label continuation of the trial indicated superior benefit of high-dose niacin supplementation on the same markers, suggesting that more research of this supplement is warranted. The limited investigation of other B-vitamins limits the conclusions that can be drawn as disease-modifying supplements in PD. Future work should explore this area more comprehensively.

### Vitamin D

The mechanism by which vitamin D may exert effects in PD remains unclear, though several have been proposed, including antioxidant and anti-inflammatory functions, as well as promotion of growth factor production.^36^ Evidence from clinical trials, on the other hand, are inconsistent. One RCT investigated a nutritional formula composed of 20 g whey protein, 2.8 g leucine, 9 g carbohydrates, 3 g fat and 800 IU vitamin D per day for 30 days in 75 participants with PD or parkinsonism.^37^ Despite optimal adherence to the dietary supplement, in conjunction with multi-disciplinary care, including physical and speech therapy, there were no between-group differences in total UPDRS score.^37^ In contrast, Suzuki et al.^38^ reported significant between-group differences in H&Y stage following 12 months of vitamin D supplementation at 1200 IU/d. Interestingly, total UPDRS score did not differ between groups, despite UPDRS generally being the more sensitive clinical measure.^38,39^ Additional evidence stems from a study of PD participants who underwent deep brain stimulation (DBS), in which high dose vitamin D, titrated according to body mass index (BMI), was administered for 12 weeks. No significant changes were observed in the levels of the pro-inflammatory biomarker C-reactive protein (CRP),^40^ although interpretation is limited by the non-specific nature of CRP which is influenced by a wide range of physiological conditions. Given the limited and inconsistent trial data, firm conclusions regarding the disease-modifying potential of vitamin D in PD cannot yet be drawn. Future trials should incorporate BMI-adjusted dosing^41^ since overweight and obese individuals require higher doses to maintain adequate vitamin D status, and include pathophysiological biomarker assessments alongside clinical outcomes.

### Vitamin E

Only one large RCT^42^ has examined the neuroprotective effects of vitamin E in PD. In a 2 by 2 factorial design, 800 PD participants were randomised to either placebo, active tocopherol and deprenyl placebo, tocopherol placebo and active deprenyl or both active drugs. At 2 years, no benefits of vitamin E were seen in UPDRS total score. Following these results, subsequent trials have evaluated vitamin E only in combination with other supplements, such as *n*-3 fatty acids. Although the rationale for this shift is unknown, it is likely that the absence of benefit in this larger monotherapy RCT contributed to vitamin E being explored primarily as an adjunct intervention, as discussed in a previous paragraph.

### Creatine

Creatine is a high energy compound which supports ATP availability through the creatine kinase system.^43^ Supplementation has been shown to enhance cognition in healthy adults, likely by buffering energy pools when metabolic demands are greater.^44^ However, evidence from clinical trials has consistently demonstrated no effect of creatine supplementation on PD progression.^45–47^ Bender and colleagues^45^ randomised participants to receive either creatine or placebo for two years and reported no significant between-group difference in UPDRS total scores. The same RCT undertook SPECT imaging to assess changes in dopamine transporter density, as a proxy measure of dopaminergic degeneration. Similar to the UPDRS scores, no differences were observed between the intervention and control groups. Another RCT^46^ by NINDS-NET PD investigators found that creatine could not be classified as futile after 12 months of 10 g/day supplementation, thus warranting further investigation into creatine supplementation. As a result, nearly 1000 participants were randomised to receive 10 g/day creatine or placebo for 5 years; however, this trial was terminated early due to a lack of efficacy in slowing the progression of PD in response to creatine.^47^ Although published in 2015, this is the most recent trial to investigate creatine supplementation in PD. Therefore, all RCTs conducted to date do not support the use of creatine as a disease-modifying therapeutic for PD.

### Coenzyme Q10

Coenzyme Q10 (CoQ10), a key component of the electron transport chain in the mitochondria, acts as a carrier of electrons and exerts antioxidant effects, protecting mitochondrial membranes from oxidative damage.^48^ It is through these antioxidant mechanisms that CoQ10 is hypothesised to decelerate PD progression. In fact, CoQ10 has been shown to be deficient in PD participants, particularly in the cerebellum, whereas sufficient levels are observed in the basal ganglia and cortex.^49^

Six RCTs^50–55^ involving over 1000 participants have investigated the effect of CoQ10 supplementation on PD progression yielding mixed findings. Shults et al.^50^ demonstrated a statistically significant reduction in total UPDRS score compared to placebo after 1200 mg/day supplementation for 16 months, whereas the H&Y score remained similar between groups. Furthermore, the same RCT investigated the effects of CoQ10 supplementation on mitochondrial function and reported improved Complex I/III activity, providing a mechanistic basis for the observed clinical improvements. While two other RCTs^51,52^ could not replicate these findings and reported no between-group differences in UPDRS total score, the latter trial by Storch and colleagues^52^ did find statistically significant between-group improvements in H&Y scores. The two largest RCTs,^50,51,55^ which assessed futility, also produced conflicting findings. These discrepancies might stem from the different sample sizes and study durations, with the lengthier and larger trial by Beal et al.^55^ establishing CoQ10 as futile. Additional heterogeneity in findings may be as a result of differences in clinical subgroups of PD, as demonstrated by Yoritaka et al.,^54^ who found that only the “wearing off” subgroup, and not the “without levodopa” subgroup, showed significant improvements in total UPDRS scores. Finally, an open-label, one-arm, dose-escalation trial by Seet and colleagues^56^ indicated that while supplementation up to 1200 mg/d of CoQ10 could reduce oxidative stress markers and boost antioxidative defences, higher doses may increase oxidative damage. However, these findings are yet to be confirmed by a larger, more robust clinical trial. The conflicting findings from RCTs has resulted in CoQ10 being classified, at the time of writing, as “non-efficacious” for delaying the progression of PD by both the International Parkinson & Movement Disorders Society^57^ and the National Institute for Health Care Excellence.^58^

### Inosine

With oxidative stress being a pathophysiological component of PD, the supplementation of inosine to increase urate levels, which is a potent antioxidant, has been investigated in PD. Two RCTs^59,60^ evaluated the effects of inosine, one of which was a dosing trial involving 75 participants for 24 months and one-month of washout. This trial reported that inosine was safe, while the secondary outcomes of efficacy demonstrated that inosine could not be labelled as futile.^59^ However, when a larger RCT was conducted with 293 PD participants, no significant difference were seen between groups in total MDS-UPDRS score.^60^ Furthermore, a higher risk of developing kidney stones was observed in the intervention group.^60^ Considering its limited efficacy and mixed safety profile, inosine may not be an intervention worth further investigation.

### Curcumin

Curcumin, the active compound in turmeric, is a polyphenol within the diarylheptanoid class and possesses potential antioxidant, anti-inflammatory and neuroprotective properties.^61^ To date, only 1 human trial has investigated the effects of curcumin on PD progression. This pilot trial by Godsi et al.,^62^ which supplemented 44 PD participants with 80 mg/d curcumin delivered via nanomicelles, was unable to demonstrate any disease-modifying effects, with no significant improvements in total MDS-UPDRS or H&Y scores. Given the limited human evidence, it remains unclear whether curcumin can alter the disease progression of PD. Larger, well-designed RCTs incorporating both pathophysiological biomarkers and clinical outcomes are needed.

### Biotics

There is compelling evidence of gut microbiota dysbiosis in PD, characterised by a reduced abundance of short chain fatty acid (SCFA) producing bacteria,^63^ which play an important role in regulating the integrity and permeability of the gut barrier.^64–66^ A decline in these species may facilitate the translocation of *α*-synuclein across the intestinal wall, thus promoting propagation of the misfolded protein.^67^ Furthermore, gut dysbiosis and increased intestinal permeability are positively associated with a pro-inflammatory state, potentially contributing to another pathophysiological hallmark of PD through the gut-brain axis.^64–66^ Dysbiosis also alters the metabolic output of the gut microbiota, shifting production towards more detrimental metabolites capable of exerting local as well as negative systemic effects, ultimately influencing brain health.^64–66^ Given its involvement in multiple systems and processes relevant to PD, the gut microbiome is increasingly regarded as an emerging hallmark of the disease. Therefore, modulation of the gut microbiome through biotics, namely probiotics, prebiotics, synbiotics, and postbiotics, may represent a means of modifying disease progression.^67,68^

Despite heterogeneity in their formulations, probiotics have shown efficacy for reducing constipation and accompanying gastrointestinal symptoms in PD,^69^ whereas evidence regarding their capacity to modify disease progression remains more scarce and inconsistent. However, a growing number of clinical trials, 7 to date, have investigated the effects of probiotics on PD-related clinical outcomes and pathophysiological biomarkers. Administration of probiotics in other inflammatory conditions, such as coeliac disease and obesity, has been associated with reduced disease morbidity and ameliorated gut inflammation,^70^ suggesting potential applicability to PD given its underlying inflammatory component. Indeed, in a 2025 trial by Leta et al.^71^ which involved 68 PD participants, probiotic administration over 12 weeks significantly decreased the inflammatory biomarkers TNF-*α* and IL-6.^71^

Similarly, Borzabadi et al.^72^ demonstrated that a 12-week intervention with probiotics significantly downregulated the expression of genes related to inflammation, including TNF-*α*, IL-1, and IL-8, whereas oxidative stress biomarkers were unchanged between the two groups. Moreover, George & Iype^73^ reported a reduction in hs-CRP following probiotic supplementation compared to no changes in the placebo group. Another RCT, which combined vitamin D with probiotics, also observed significant decreases in multiple circulating pro-inflammatory cytokines alongside reductions in total UPDRS scores; however, it remains unclear how these interventions may interact synergistically or complementarily to produce the observed therapeutic effects.^74^ Du et al.^75^ additionally showed significant reductions in UPDRS scores after 12 weeks of probiotic supplementation, indicative of potential slowing of disease progression. It is noteworthy that this trial was open-label, however, limiting the robustness of these findings. In contrast, supplementation with the *Lacticaseibacillus paracasei* strain Shirota alone failed to improve total MDS-UPDRS score over 12 weeks.^76^

To date, there has been one small, single-arm, open-label, non-randomised study that assessed prebiotic fibre for its disease-modifying potential in PD.^77^ Following 10-day prebiotic supplementation, total UPDRS score decreased along with reductions in plasma levels of zonulin and calprotectin, both of which are barrier integrity and inflammation biomarkers. However, CRP and all measured serum cytokines were unchanged after supplementation, possibly reflecting the short study duration. Well-designed RCTs with larger sample sizes and longer study durations are needed to better understand how prebiotics may change pathophysiological biomarkers and clinical progression of PD.

While there have been no trials investigating postbiotics for PD, synbiotic interventions, which combine probiotics and prebiotics, have been explored. In a 12-week RCT including 80 PD participants, synbiotic supplementation increased total antioxidant capacity relative to placebo.^78^ These findings were confirmed by an open-label study of similar duration, which documented decreases in malondialdehyde, an oxidative stress biomarker, and TNF-*α.*^79^ Both biomarkers were strongly correlated with improvements in Parts I, II and III of the MDS-UPDRS, though part IV was not measured and therefore changes in disease progression could not be directly attributed to pathophysiological biomarker alterations. Magistrelli and colleagues^80^ randomised participants to receive either a synbiotic formulation or placebo and assessed changes in clinical outcomes, including total UPDRS and H&Y scores, as well as circulating cytokines. While reductions in IFN-*γ* and IL-6 in the intervention group were described, no between-group differences were reported. All remaining outcomes that were evaluated were unchanged in both groups.

Six trials investigated the gut microbiome following probiotic, prebiotic, or synbiotic supplementation. Although each trial reported changes in specific bacterial taxa likely reflecting the diversity of biotic formulations used in trials, the functional changes were largely consistent. All trials except Yang et al.^76^ observed changes suggestive of improved gut microbial function, including increases in bacterial taxa that produce SCFAs,^71,77,81,82^ or reductions in genera elevated in PD and considered detrimental, including mucin-degrading *Prevotella* and pro-inflammatory Proteobacteria.^75,77,82^ These findings support the hypothesis that, despite substantial inter-individual variability in gut microbiota composition, functional redundancy across microbial species may make compositional changes alone less informative.^83^ In contrast, Yang et al.^76^ reported only a significant increase in *Lacticaseibacillus paracasei* Shirota, the supplemented strain, without broader functional shifts. Interestingly, this was the only probiotic RCT that did not demonstrate improvement in total MDS-UPDRS score, raising the possibility that single-strain probiotics may be less effective than multi-strain formulations. The only other study to use a single-strain, Sun et al.^82^ did not assess clinical progression or other pathophysiological biomarkers, limiting conclusions.

Importantly, a meta-analysis of 11 RCTs reported minimal adverse effects of probiotics across heterogeneous PD populations, underscoring their favourable safety profile.^84^ As the gut microbiome composition and function are increasingly recognised as being correlated with numerous PD pathophysiological hallmarks, including *α*-synuclein aggregation, inflammation, and oxidative stress,^85^ biotics represent a promising area for future investigations into their potential disease-modifying effects.

## Future perspectives

A range of potential disease-modifying dietary supplements for PD have been proposed. Whilst several trials have reported encouraging results, there are some supplements that may not warrant further investigation. Given the lack of efficacy coupled with an increased risk of adverse events, the supplement inosine will unlikely be considered for future trials. Similarly, coenzyme Q10 has largely yielded inconsistent findings leading to a formal classification of non-efficacious. The early termination of the largest trial of creatine in PD has indicated futility of the supplement in delaying disease progression. There is currently insufficient evidence on other supplements to draw definitive conclusions. In contrast, the most promising candidates worth pursuing appear to be B-vitamins, biotics and *n*-3 fatty acids, although more robust clinical research is required to substantiate preliminary insights (Figure 1).

Looking ahead, two major trials of NR have recently been completed but no results have been reported yet. NOPARK (NCT03568968) is a double-blind phase II RCT that administered 1000 mg/day for 52 weeks, enrolled 400 participants and is designed to transition participants into receiving an open-label extension in which the dosage will be increased to 1200 mg/day with annual follow-up for 3 years. N-DOSE (NCT05589766) is a double-blind, placebo-controlled RCT that aims to examine whether escalating NR doses beyond 1000 mg/day provides additional benefit, employing a 12-week protocol with doses rising from 1000 mg to 3000 mg/day in 81 participants. Together, these two trials represent the largest NR trials to date and might help clarify whether NR can emerge as a viable candidate for disease modification.

Biotics continue to play an important role in PD research yet several aspects of biotic interventions remain unexplored.^64^ Importantly, no biotic has been approved by the FDA or EFSA as a therapeutic agent, and widespread clinical implementation thus awaits more robust evidence of efficacy. Another major challenge is the substantial heterogeneity in probiotic formulations. This variability can lead to distinct effects on different metabolic pathways even amongst closely related microbial taxa, as strains belonging to the same genus and species may differ markedly in function.^86^ Thus, identifying which bacterial strains exert effects most relevant to PD remains an essential research priority. Further variability exists in delivery formats and doses of administration, often without strong rationale for differences between trials. Modes of delivery range from fermented milk products to capsules and sachets, and dosages can vary by over 100-fold across trials. Despite numerous investigations into biotics for gastrointestinal symptoms and potential disease modification, the determinants of strain survival through the gastrointestinal tract and their ability to colonise the gut remain poorly understood, particularly across different modes of administration.^87^ Moreover, interactions between probiotic strains could lead to synergism or antagonistic effects due to nutrient competition in the gut.^88^ *In vivo* research is needed to clarify these dynamics and optimise multi-strain formulations while minimising unintended side-effects. Future human trials should additionally seek to understand how different biotics influence PD across different stages of pathology, investigating not only gut microbiome composition but also microbial function, pathophysiological pathways, and clinical outcomes. Advances in biomarkers of disease progression may help overcome limitations of existing clinical measures, enabling more sensitive detection of disease-modifying effects.^89^ Larger, multi-arm and multi-centre RCTs with longer follow-up periods are required to determine whether the observed microbiome shifts persist, particularly given the well-documented resilience of the gut microbiome and their tendency to return to baseline after supplementation ceases,^90^ as seen in some fecal microbial transplant research.^91^ Long term trials and follow-up studies are therefore essential to understand the dynamic nature of the PD gut microbiome. Ultimately, improving our mechanistic understanding of microbiome-host interactions may pave the way for personalised probiotic strategies tailored to an individual's microbiome composition. In addition, next-generation probiotics,^92^ compromising non-conventional strains with mechanistic relevance, such as *Faecalibacterium prausnitztii*, which has been reported to be reduced in PD,^93^ represent a promising avenue for future exploration.^94^

Despite conflicting results in clinical outcomes following the combined supplementation of *n*-3 fatty acids and vitamin E, the promising reductions in pro-inflammatory biomarkers support potential disease-modifying properties. The findings from Neuroaspsis supplementation reflects the importance of combining interventions to synergistically target different disease-modifying mechanisms. Indeed, a holistic approach is likely to be most effective, and future research should combine several interventions into integrated lifestyle programmes to target multiple pathophysiological mechanisms of PD simultaneously. Diet and exercise have shown synergistic benefits in other chronic inflammatory conditions,^95^ and this may extend to PD.^10^ Considering the heterogeneity of PD clinical phenotypes, combining interventions increases the probability of influencing differing disease trajectories whilst supporting more comprehensive patient management.

The transition from animal studies to human trials remains another key barrier in the development of interventions that may be beneficial in PD. Indeed, over the years, many compounds have shown promise *in vivo* yet subsequently failed to translate into human benefits. Nonetheless, several nutraceutical compounds with encouraging preclinical evidence have not yet been trialled in humans.^96,97^ For example, flavonoid-containing supplements such as baicalein have demonstrated reductions in neuroinflammation, oxidative stress and mitochondrial dysfunction in mouse models.^98,99^ Epigallocatechin gallate, another potent flavonoid, has also shown protective effects against *α*-synuclein aggregation and modulatory effects on antioxidant signalling pathways in both *in vitro* and *in vivo* studies.^100^ Beyond flavonoids, carvacrol which is a phenolic compound, is another promising candidate for human research as it has exhibited antioxidant and anti-inflammatory properties *in vivo.*^101^ Given the promising disease-modifying effects observed in pre-clinical studies, nutraceuticals warrant further investigation in human studies to assess their effects in PD.

Continued research into potential disease-modifying dietary supplements and dietary strategies, across *in vitro*, *in vivo* and human trials is imperative for advancing the future therapeutic options in PD. Importantly, recognising disease-modifying potential, whether through mechanistic biomarkers or clinical endpoints, is only the first step. To confirm true disease modification, trials could incorporate delayed-start designs similar to the ADAGIO and TEMPO trials on MAO-B inhibitors^102,103^ to determine whether early-treated groups ultimately fare better than delayed-treatment groups. Likewise, washout designs can be used to assess whether therapeutic withdrawal preserves any sustained benefit as implemented in the ELLDOPA RCT.^104^ These approaches help to establish whether a therapy exerts effects beyond symptomatic improvement. Long term follow-up is also crucial to verify that clinical milestones are consistently delayed. Thus, even for promising dietary supplements such as NR, *n*-3 fatty acids and biotics, improvements in delayed-start or washout trial designs must be seen before disease modification can be confirmed. This could be the aim of future research once these interventions have demonstrated more robust disease-modifying potential than is currently available.

## Conclusion

Whilst the search for disease-modifying and curative therapies continues, the management of PD remains of upmost importance. Dietary supplements hold promise in disease modification, with biotics, nicotinamide riboside, and omega-3 fatty acids emerging as key candidates for future research. Ongoing research within this area is of essence given the high prevalence of dietary supplement use in PD, despite the absence of robust evidence supporting disease-modifying benefits. Strengthening the evidence will enable clinicians to offer more informed recommendations on dietary supplement use. It is equally important to emphasise that managing nutrient deficiencies remains a vital component of PD care due to the prevalence of such deficiencies. Future research should aim to clarify when, how, and for whom specific dietary supplements provide meaningful benefit, ultimately supporting more personalised and effective management for PD participants.

## Take-home messages

- Dietary supplements may have disease-modifying potential.
- Biotics, nicotinamide riboside and the combination of *n*-3 fatty acids and vitamin E currently demonstrate the most promise and should be the focus of future research.
- A multidisciplinary approach should be taken when planning trials, including measurements of a variety of clinical and pathophysiological biomarker outcomes.
- Larger, long term, methodologically robust trials and relevant follow-ups are needed to draw more definite conclusions.
